# Radiology in Brazil and the global resolution on strengthening medical imaging: a regional responsibility

**DOI:** 10.1590/0100-3984.2025.0106

**Published:** 2026-02-27

**Authors:** Rubens Chojniak, Giovanni Guido Cerri

**Affiliations:** 1 Brazilian College of Radiology and Diagnostic Imaging, São Paulo, SP, Brazil; Department of Imaging, A.C. Camargo Cancer Center, São Paulo, SP, Brazil.; 2 The Lancet Oncology Commission on Medical Imaging and Nuclear Medicine, London, UK; Universidade de São Paulo Hospital das Clínicas Institutes of Radiology (InRad) and Innovation (InovaHC), São Paulo, São Paulo, SP, Brazil.

**Keywords:** Diagnostic imaging, Delivery of health care/organization & administration, Health personnel/organization & administration, Global health, Health policy, World Health Organization, Radiology/statistics & numerical data, Brazil, Diagnóstico por imagem, Atenção à, saúde/organização & administração, Pessoal de saúde/organização & administração, Saúde global, Política de saúde, Organização Mundial da Saúde, Radiologia/estatística & dados numéricos, Brasil.

## Abstract

In May 2025, the World Health Assembly adopted Resolution WHA78.13, formally recognizing medical imaging as a fundamental pillar of universal health coverage. Brazil, as a co-sponsor of the Resolution, plays a decisive role in promoting equitable access to imaging services throughout Latin America. This article explores the implications of the resolution for Brazil, highlighting its leadership and the need to address regional disparities in imaging infrastructure and workforce distribution. On the basis of data from the 2025 Atlas of Radiology in Brazil, the article highlights the unequal distribution of radiologists and imaging equipment, especially in underserved areas. Strategic priorities for regional action are outlined, including workforce development, infrastructure investments, and public policy integration. The Brazilian College of Radiology and Diagnostic Imaging is positioned to participate in national efforts, advocating for medical imaging as a public health priority. By aligning national initiatives with global strategies and embracing innovation, Brazil and Latin America can transform diagnostic equity and improve health outcomes.

## Abstract (pt)

Em maio de 2025, a Assembleia Mundial da Saúde adotou a Resolução WHA78.13, reconhecendo formalmente a imagem médica como um pilar fundamental da cobertura universal de saúde. O Brasil, como co-patrocinador da resolução, assume um papel decisivo na promoção do acesso equitativo aos serviços de imagem em toda a América Latina. Este artigo explora as implicações da resolução para o Brasil, destacando sua liderança e a necessidade de enfrentar as disparidades regionais na infraestrutura de imagem e na distribuição da força de trabalho. Com base em dados do *Atlas da Radiologia no Brasil 2025*, o artigo evidencia a distribuição desigual de radiologistas e equipamentos de imagem, especialmente em áreas carentes. São delineadas prioridades estratégicas para ação regional, incluindo o desenvolvimento da força de trabalho, investimentos em infraestrutura e integração de políticas públicas. O Colégio Brasileiro de Radiologia e Diagnóstico por Imagem está posicionado para participar dos esforços nacionais, defendendo a imagem médica como uma prioridade de saúde pública. Ao alinhar iniciativas nacionais com estratégias globais e adotar a inovação, o Brasil e a América Latina podem transformar a equidade diagnóstica e melhorar os desfechos em saúde.

## INTRODUCTION

In May 2025, the World Health Assembly (WHA) approved the historic Resolution WHA78.13 on strengthening medical imaging capacity^(1)^. For the first time, medical imaging was formally recognized as an essential pillar of universal health coverage.

The Resolution emphasizes the need for countries to develop strategies in workforce training, infrastructure, regulation, and access to safe, high-quality imaging, acknowledging its critical role in diagnosing, treating, and monitoring communicable and noncommunicable diseases. Brazil, alongside Cameroon, Armenia, and Burkina Faso, played a central role as a co-sponsor of the WHA Resolution. This leadership reflects the national and regional responsibility to strengthen access to medical imaging, given that Latin America continues to face profound inequities: while large urban centers are equipped with advanced modalities, rural and underserved areas still lack basic diagnostic tools such as ultrasound and radiography.

Closing this gap is not only a public health priority but also an ethical imperative.

### The Brazilian context

Noncommunicable diseases, including cancer, cardiovascular disease, and stroke, account for nearly three-quarters of premature deaths globally, with the heaviest toll in low- and middle-income countries. In Brazil, these conditions remain the leading causes of mortality and disability. Without timely access to imaging, early cancer detection, acute stroke intervention, or effective heart disease management cannot be delivered at scale.

Evidence from the Lancet Oncology Commission on Medical Imaging and Nuclear Medicine^(2)^ demonstrates that scaling up imaging capacity could prevent millions of deaths and yield a significant return on investment, estimated at US$12.43 per dollar spent. Such data must guide national and regional health policies in Latin America, where inequity in diagnostic access continues to compromise outcomes.

### Opportunities for regional action

The WHA Resolution creates an unprecedented opportunity for Latin America to act collectively. Three priority areas stand out:

1. **Workforce development —** Expansion of training programs for radiologists, radiographers, and physicists is critical. Partnerships with universities, professional societies, and international organizations such as the International Atomic Energy Agency and World Health Organization are essential to scale education and accreditation.
2. **Infrastructure and technology —** Investment must include durable, cost-effective equipment, mobile imaging units for rural areas, and digital platforms linking imaging to national health records. Artificial intelligence offers unique opportunities to democratize expertise, bringing diagnostic accuracy to underserved regions^(3)^.
3. **Policy and financing —** Imaging must be integrated into cancer control plans, essential diagnostics lists, and universal health coverage frameworks. Innovative financing models, public–private partnerships, regional procurement strategies, and outcome-based funding are necessary to ensure sustainability^(4)^.

### A call to leadership

As co-sponsor of the WHA Resolution, Brazil has the responsibility to ensure its principles are effectively translated into action. The upcoming 2026 International Congress of Radiology, in Cartagena, Colombia, will provide a regional platform to discuss national implementation strategies and inspire collective action across Latin America.

The *Colégio Brasileiro de Radiologia e Diagnóstico por Imagem* (CBR, Brazilian College of Radiology and Diagnostic Imaging), in partnership with allied societies, must advocate strongly for imaging as a central component of healthcare planning. Beyond technological advances, this is a matter of equity and social justice; a patient’s social condition should never determine their access to life-saving medical imaging.

### Insights from the 2025 Atlas of Radiology in Brazil

Recent demographic data from the 2025 Atlas of Radiology in Brazil^(5)^, published by the CBR, reveal the strengths and inequities in the national distribution of radiologists and imaging resources.

According to the Atlas, there are 20,453 certified radiologists in Brazil, representing approximately 3.2% of all medical specialists in the country. However, the density of radiologists is uneven: the average number of radiologists per 100,000 population is 13.18 in the southeast region, compared with fewer than 5 in the north and fewer than 8 in the northeast. Nationally, the average is 10.87 radiologists per 100,000 population. This gap translates directly into disparities in access, with the density of radiologists being nearly four times greater in state capitals than in smaller municipalities.

In addition to professional distribution, the Atlas highlights important disparities in the availability of imaging equipment. While high-complexity modalities such as computed tomography, magnetic resonance imaging, and positron emission tomography/computed tomography are concentrated in major urban centers and predominantly in the private sector, access through the public health care system remains markedly limited in several states and regions. This dual challenge of human resources and technological infrastructure further compounds inequities in diagnostic capacity across the country.

These figures illustrate the structural challenges that must be addressed if Brazil and Latin America are to achieve the goals of Resolution WHA78.13. The Atlas also underscores the need for policies of professional training, wider dissemination of services, and more balanced distribution of human and technological resources, ensuring equitable access to life-saving imaging technologies across all regions.

As the leading national society within the sphere of imaging, the CBR can play a decisive role in this process, not only by providing robust epidemiological data and promoting professional education but also by actively participating in, and giving support or advice to, public agencies and entities committed to expanding equitable access to imaging services.

## CONCLUSION

The adoption of Resolution WHA78.13 marks a turning point for global health policy. For Brazil and Latin America, it is both an opportunity and an obligation. By aligning national initiatives with global strategies, strengthening workforce training, modernizing infrastructure, and embracing innovation, our region can lead by example.

Radiology has the power to transform outcomes, save lives, and reduce inequities. The global consensus has been reached, and the time for implementation is now.

## Data Availability

Not applicable
